# Modeling and Simulation of Complex Network Attributes on Coordinating Large Multiagent System

**DOI:** 10.1155/2014/412479

**Published:** 2014-04-03

**Authors:** Yang Xu, Xiang Li, Ming Liu

**Affiliations:** School of Computer Science and Engineering, University of Electronic Science and Technology of China, Chengdu 611731, China

## Abstract

With the expansion of distributed multiagent systems, traditional coordination strategy becomes a severe bottleneck when the system scales up to hundreds of agents. The key challenge is that in typical large multiagent systems, sparsely distributed agents can only communicate directly with very few others and the network is typically modeled as an adaptive complex network. In this paper, we present simulation testbed *CoordSim* built to model the coordination of network centric multiagent systems. Based on the token-based strategy, the coordination can be built as a communication decision problem that agents make decisions to target communications and pass them over to the capable agents who will potentially benefit the team most. We have theoretically analyzed that the characters of complex network make a significant difference with both random and intelligent coordination strategies, which may contribute to future multiagent algorithm design.

## 1. Introduction

Multiagent system as a paradigm of distributed artificial intelligence has been popular in domains such as military, aeronautics, and disaster rescue. For example, large heterogeneous multirobot teams respond to large-scale natural disasters [1], performing future constructions and managing large civic infrastructures [2], deploying large groups of unmannered aerial vehicles and ground vehicles in military operations [3]. In those systems, the key is to build practical coordination algorithms to be able to control the heterogeneous agents toward their common objective. Although there have been many popular research works towards this issue [4], when state of the art applications require more and more agents, existing coordination approaches are not capable of addressing the new challenges of controlling hundreds of agents.

First of all, network structure has been a key attribute to the success of coordination algorithm design. For example, when a large team of robots are deployed, they are always sparsely distributed. With the restrictions of wireless communication coverage, agents may only be able to communicate directly with very a few of the others. Therefore, similar to human society dynamic infrastructure, each time, agents only closely coordinate with few teammates via communication networks either logically or physically, while they communicate with all the others with multihops of retransmission.

Secondly, multiagent systems are required to react to dynamic and partially observable environment with agents' independent decisions. Because of the nature of the multiagent application domains, agents are always in a complex, dynamically changing, and, in some cases, hostile environment. To react to the critical domain events, they have to intellectually adapt to the dynamic changes and coordinate themselves with their own observation and knowledge. In this process, network is critical to either make agreement on agents' joint activities or receive information to support agents' decisions when it cannot be completely observed.

Thirdly, coordination is a complex process since several distributed algorithms are required to interact to produce agile, cohesive, and efficient coordinated behavior. Typical coordination requires different aspects of subtasks interacted between networked agents, such as sharing information on external environmental events [5], allocating individual tasks for joint actions [6], and sharing exclusive resources [7].

The communication network is a major bottleneck to an efficient and scalable coordination. Existing algorithms for small team coordination, that is, blackboard [8] or specific centralized agents [9], relying on excessive communication protocols are either infeasible, undesirable, or too expensive in large-scale teams. To reduce communication and make the decentralized coordination possible, agents should attempt to target their communications. However, it is a chicken-and-the-egg puzzle where communication is required for sharing knowledge while rich localized knowledge is indispensable to target communications.

In summary, with the expansion of the multiagent system, network has been a key factor to model team coordination. It is indicated, in related works [11], that different topologies may significantly change the performances over the same controlling protocol. The key of simulating multiagent coordination is to solve the problem of how decentralized agents are interacting over communication network, so that different aspects of coordination tasks can be realized to reach their common goal. Although popular network simulation tools such as NS2 [12] and OPNET [13] are able to simulate the performances of typical distributed system applications, they are not able to simulate the multiagent coordination in large-scale systems. We summarize the reasons as follows.

(1) Agents use proactive decision model to interact with others and dynamically choose who to communicate with, instead of traditional predefined reactive communication protocol.

(2) Agents are required to build local knowledge base and dynamic reasoning model to make decentralized coordination decision, which are not possible to be encoded in those network simulation tools.

(3) Agents are required to communicate many complex semantic data such as domain information, tasks, and sharable resource with others. But these data cannot be uniformly abstracted as communication packages as most network simulators do.

Although there have been existing multiagent team coordination simulators, most of them cannot simulate the large-scale networked multiagent system well. For example, Machinetta [14] that simulated the multiagent coordination in real domain, ignored the complexity of network structure and only modelled agents in a small team with a complete network. Framework for task analysis, environment modeling, and simulation (TAEMS) [15] uses a partial global planning (PGP) framework [16] for domain-independent decision making in handling uncertainty in the environment. It is built based on a hierarchical network architecture. Other decentralized coordination simulators, such as LA-DCOP [17], are only capable of partial coordination subtasks. In this paper, we will analyze the characters of complex networks which will make a significant difference in the multiagent coordination, and based on these observations, we put forward a novel approach to model and simulate coordination in networked multiagent systems. The simulator is capable of simulating the major aspects of coordination including information sharing, task assignment, and resource allocation. The key is to simulate how agents interact over the network so that agents can carry out their joint activity toward their common objective. The most prominent advantage of the simulator is that it allows a large number of parameters critical to the coordination to be adjusted and also allows statistics to be recorded; especially, it is able to initiate the agents in different network topologies by taking the complex network effects into consideration. These variations and records are helpful to verify the efficiency of coordinating large-scale networked multiagent systems.

## 2. Coordination for Multiagent System

The coordination for a multiagent system *A* = {*a*_1_, *a*_2_,…, *a*_*n*_} can be described as follows. Networked agents share a top level common goal Goal (as in [18]), and achieving Goal requires achieving a number of subgoals {*g*_1_, *g*_2_,…, *g*_*i*_,…}. When subgoal *g*_*i*_ is satisfied, the team will receive a reward reward_*i*_. For example, subgoals of a high level goal to respond to a disaster might be to extinguish fires and provide medical attention to injured civilians. To satisfy subgoals, the team follows a set of Plan = {plan_1_, plan_2_,…, plan_*i*_,…} represented in a library [19]. Each plan *i* includes four parts and is written as plan_*i*_ = 〈*g*_*i*_, conditions_*i*_, tasks_*i*_, reward_*i*_〉. The first element is the subgoal *g*_*i*_; the second is the conditions under which it is applicable, conditions_*i*_ = event_1_∩event_2_∩⋯∩event_*l*_; the third element is the individual tasks tasks_*i*_ = {*r*_1_, *r*_2_,…, *r*_*k*_} which are required to achieve *g*_*i*_ and the last part, the reward_*i*_, is to be received by the team on successful satisfaction of *g*_*i*_. Each task *r*_*i*_ = 〈*g*_*i*_,  capability_*i*_,  res_*i*_〉 is represented by its subgoal *g*_*i*_ carried out by a single agent with the required certain kind of capability_*i*_ and res_*i*_ (which would be introduced below), that is, a description of the actual things for the agent to do to realize the subgoal *g*_*i*_.

For example, a fire fighting plan can be modeled as follows: plan_fire_ = 〈(Fight  fire  at  location  *X*), (Fire  alarm  at  *X* ∩Smoke  at  *X*), {*r*_1_, *r*_2_, *r*_3_}, (100)〉. This plan requires two conditions before it is initiated: a fire alarm and smoke. After this plan is initiated, three tasks, {*r*_1_, *r*_2_, *r*_3_} need to be assigned and a reward 100 will be credited to the team. The three tasks in the plan are driving the fire truck, fighting the fire, and searching for victims; that is, *r*_1_ = 〈(driving  the  fire  truck), (skillful in driving truck), (fire  truck)〉, *r*_2_ = 〈(fighting  the  fire), (have training in fire fighting), (hose, water)〉, and *r*_3_ = 〈(searching  for  victims), (none), (breathing  equipment)〉. To perform *r*_1_, an agent is required to be able to drive and have access to a fire truck which is an exclusive resource.

In a heterogeneous multiagent system, each agent may have different capabilities to perform different tasks. For example, in the fire rescuing, the capable agents may be able to drive the fire truck, fight fire, or search for the wounded. However, when performing the same task, agents with different capabilities make different performances; for example, a fireman is more capable than an inexperienced citizen in a fire fighting. Therefore, we define a function capability(*a*_*i*_, *r*) to denote the capability of agent *a*_*i*_ to perform task *r*. If the agent's capability is more than *r*'s threshold, it is able to perform the task. A reward will be received by the system when an agent finishes a task. It is a function of an agent' capability and task, as well as the resources the agent has. Specifically,

(1) $$\begin{array}{c} \text{reward}\left(a_{i},r,\text{Holds}\left(a_{i}\right)\right)⟶ℛ. \end{array}$$

The function Assigned(*a*_*i*_, *r*) = 1 if agent *a* is assigned to task *r*; otherwise it is equal to 0. At any time, a task could only be assigned to at most one agent; that is, ∑_*a*_*i*_∈*A*_Assigned(*a*_*i*_, *r*) ≤ 1.

Networked agents always require sharable resources to perform tasks. These resources, *R* = {res_1_,…, res_*m*_}, are discrete and nonconsumable. Agent *a*_*i*_ has exclusive access to resources Holds(*a*_*i*_)⊆*R*. Only one agent may hold a resource at any point of time; that is, for  all  *a*_*i*_, *a*_*j*_ ∈ *A*, *a*_*i*_ ≠ *a*_*j*_, Holds(*a*_*i*_)∩Holds(*a*_*j*_) = *∅*.

The coordination problem is to maximize the rewards to the team, while minimizing the costs of coordination. The overall reward is simply

(2) $$\begin{array}{c} \underset{a_{i}\in A}{\sum }\underset{r_{i}\in {\text{task}}_{i}}{\sum }\text{Assigned}\left(a_{i},r_{i}\right)\text{Reward}\left(a_{i},r_{i},\text{Holds}\left(a_{i}\right)\right). \end{array}$$

The costs of coordination can be very general and in some cases difficult to define. Here we are specifically concerned with only the volume of communication.

## 3. Networked Agents

**Figure 1:**
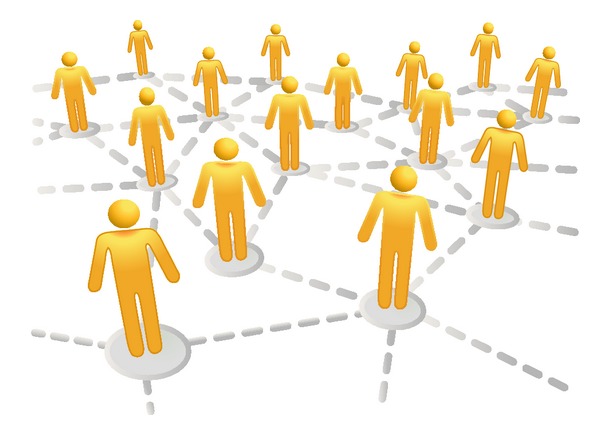
An example of large-scale multiagent network.

According to the characters of networked agents that they can only directly interact or communicate with very few of others over the network, the organization of the multiagent system can be abstracted as an undirected network *G* = (*A*, *E*) as shown in Figure 1 [20]. In this model, *A* is the agent set, and *E* is the set of edges where if *e*_*k*_ = 〈*a*_*i*_,  *a*_*j*_〉, *a*_*i*_ and *a*_*j*_ can communicate with each other directly. Moreover, *n*(*a*_*i*_) is defined as the set of *a*_*i*_'s neighbors that it can directly communicate with.

*G* could be organized as different network topologies based on the different properties of social networks. In our simulation, we are mainly interested in four of them: random network, grid network, small world network, and scale-free network. Preliminary studies [21] found that each topology encodes the following different fundamental properties.

1. Average degree: $\bar{d}=\left(1/N\right)\sum_{a_{i}\in A}|n(a_{i})|$.
2. Degree distribution: *p*(*k*) = Pr [*d* = *k*].
3. Average distance:*d*_ave_ = (1/*N*(*N* − 1))∑_∀*a*_*i*_,*a*_*j*_∈*A*_distance (*a*_*i*_, *a*_*j*_). distance(*a*_*i*_, *a*_*j*_) defines the least number of hops to communicate between *a*_*i*_ and *a*_*j*_.

Different network topologies can be described according to the properties above. In our approach, we mainly take consideration of the effects of small world and scale-free network and use degree distribution to express the scale-free effect and their average distance to express the small world effect.

## 4. Networked Coordination Model

As the coordination problem modeled in Section 2, agents' interactions over network are to carry out different aspects of coordination subtasks to reach their common goal including information sharing, task allocation, and resource sharing. INF = {event_1,1_^1^, event_1,1_^2^,…, event_*i*,*j*_^*k*^,…} is the set of all possible domain events, TASK = {task_1,1_^1^, task_1,1_^2^,…, task_*i*,*j*_^*k*^,…} is the set of potential available joint activities, and RESOURCE = {res_1_, res_2_,…, res_*k*_,…} is all available exclusive resources in team *A*. Multiagent coordination is defined as *Ξ* = INF ∪ TASK ∪ RESOURCE. At each time, the coordination element interacted between a pair of agents over the network can be defined as *tc* ∈ *Ξ*, which is either a domain event, a joint activity, or an exclusive resource. By encapsulating* tc*, we can define the data structure of the interaction between agents in the network as Token.

If we assume that agents will handle their own jobs well, the objective of communicating token through networked agents is to find capable agents who can perform the desired task, use resource, or obtain the information, so that a subgoal *g*_*i*_ will be achieved. The structure of any token Δ_*j*_ is written as Δ_*j*_ = 〈ID, *tc*, path, threshold〉. We assume that token cannot be duplicated. When an agent is holding Δ_*j*_, it takes over control Δ_*j*_.*tc* and will release Δ_*j*_.*tc* if it is passed. Δ_*j*_.path records the sequence of agents where Δ_*j*_ has been passed. Δ_*j*_.path is also used as stop condition for information and task tokens if |Δ_*j*_.path|>Length. Length is predefined that Δ_*j*_ is allowed to be passed to others before being stopped.

Threshold generalizes a threshold for resource and task tokens that can be accepted by any agent. Only when the capability or calculated desire of an agent is higher than Δ_*j*_.threshold, it can be accepted. Threshold can be dynamically adjusted to balance the needs of the whole system and produce improved allocation performance. For example, if a resource is highly competitive, it will enhance the threshold to travel across the network to find a “better” accepter. Otherwise, if it has traveled a long way over the network, the threshold should be decreased to help to find anyone who can make use of it, even if it may not be the best.

## 5. Multiagent Communication Decision

As explained in Section 4, the objective of communicating token is to find capable agents who can perform the desired task, use resource, or obtain the information if themselves are not capable of making use of it. Similar to human society, agents always forward the incapable tasks and resources across the network to find the best accepters. The key of the coordination is to optimize their interactions, so that the best capable agents could be reached as fast as possible with the least communication cost as well as assignment delay.

Algorithm 1 [22] describes the general decision process for decentralized networked agents. In this algorithm, agents first check whether new tasks have become applicable. If so, the agent will embed the task to a token and add it into its token list Tokens, which contains the tokens to be processed (lines 3–7). Next, the agent will receive all the tokens passed from other agents (line 8). It then processes all the tokens in the Tokens. If a token represents a task, the agent will accept the task if its capability to perform that task is higher than token's threshold (lines 11–14); otherwise, the agent will choose a neighbor to pass that token to (line 17). If the token is a resource token and the agent's need for that resource to perform his waiting tasks is higher than token's current threshold, this resource will be held; otherwise, it is passed to a neighbor (lines 19–27). Note that when a token is sent, it will be removed from that agent's list. Finally, the agent will check whether any task which is appended can now be executed (lines 30) and release any resources from completed tasks (lines 32–37).

The key of the decision process is the function of SendToNeighbour( ), which is to pass a token to a neighbor who either needs it or knows who does. In our simulation model, we primarily use two algorithms in which tokens are routed either intelligently or randomly. By building a local decision matrix of an agent *a* for a token Δ as *P*_*a*_[Δ]. *P*_*a*_[Δ, *b*]→[0,1], *b* ∈ *n*(*a*) represents *a*'s decision of the probability of passing token Δ to neighbor *b*. In random routing algorithm, for each *b* ∈ *n*(*a*), *P*_*a*_[Δ, *b*] is uniform. However, when agent *a* has built a knowledge base for its received tokens, agent *a* can route Δ in a smart way. By taking the advantage of tokens that may be related in domain knowledge, previous received tokens can be used to update local probability model that receiving a token predicts that the sender may request a related token. For example, the sender of the information token “I am hungry” denotes that it may be interested in a resource token “pizza” to feed a hungry agent. Therefore, each token is used to improve the routing of other tokens which will lead to a dramatic performance improvement. The key algorithm has been detailed and described in [23] and has been encoded in our simulator.

## 6. Complex Network Effects in Coordination Efficiency

In this section, we briefly analyze how complex network effects will change the team coordination performance. We modeled the token's random walk as a finite Markov chain and it is time reversible [24]. For a specific token movement, we can define different states.

**Figure 2:**
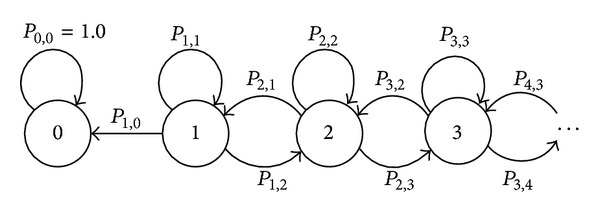
Markov chains model on tokens' movement between agents.

As it is shown in Figure 2, state *s*_*i*_ defined the state that the token moves to an agent with the shortest distance of *i* from the sink agent. Specifically, probability *P*_*i*,*j*_ defined the probability of the token being passed from states *i* to *j*. Because the token can only move one step for each horizon, *P*_*i*,*j*_ = 0 except for *j* ∈ {*i* − 1, *i*, *i* + 1}. Therefore, for a state *s*_*i*_ ≠ *s*_0_, the token may move closer to the destination (*P*_*i*,*i*−1_), stay on the same level (*P*_*i*,*i*_), or move far away (*P*_*i*,*i*+1_). When the token reaches state *S*_0_, it will be stopped at the destination and the probability *P*_0,0_ = 1. For example, suppose that *u* is the initial probability distribution of the token being in state *S*. According to the theory of Markov chains, we can calculate the probability that the token reaches the sink agent after *n* steps as *P*_*S*_^*n*^ = *u* × *P* ^*n*^.

**Figure 3:**
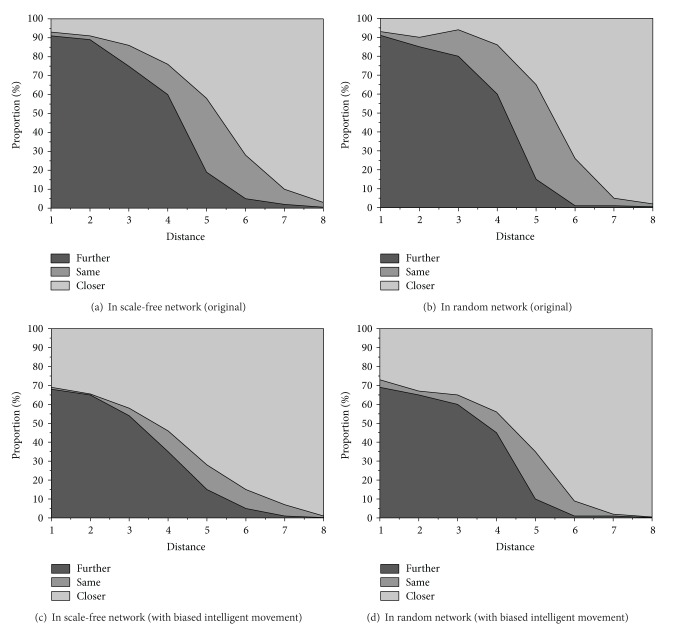
The relative proportions of links that lead closer to, keep the same distance, or move further from the target agent, as the distance to it is varied [10].

As the agents transmit the tokens randomly to anyone of their neighbors, there will be different distances between the source and destination.  Figure 3 shows the relative rates of *P*(*s*_*i*_, *s*_*i*−1_) (marked “Close”), *P*(*s*_*i*_, *s*_*i*_) (marked “Same”), and *P*(*s*_*i*_, *s*_*i*+1_) (marked “Further”) for scale-free and random networks. Notice that we average *P*(*s*_*i*_, *s*_*j*_) over each node at distance *i*, though this will vary from node to node. The *x*-axis shows the distance from a node to the target node, that is, the target agent *i*. The *y*-axis shows the proportion of the states of “Further,” “Same,” and “Closer,” different colors represent the corresponding proportions, and the sum of them is 1. Note that the closer the token is to the target, the more likely it will be randomly passed further from the target. Correspondingly, the further the token is from the target, the more likely it will be passed closer to the target in random movement. Moreover, since the figures show different distributions, their token movement characteristics are likely to be different.

Figure 3(a) shows a typical scale-free network. The state probability transition matrix *P* in this network topology is

(3) $$\begin{array}{c} \left(\begin{array}{cccccccc} 1 & 0 & 0 & 0 & 0 & 0 & 0 & 0\\ 0.1 & 0.01 & 0.89 & 0 & 0 & 0 & 0 & 0\\ 0 & 0.15 & 0.1 & 0.75 & 0 & 0 & 0 & 0\\ 0 & 0 & 0.25 & 0.25 & 0.5 & 0 & 0 & 0\\ 0 & 0 & 0 & 0.45 & 0.35 & 0.2 & 0 & 0\\ 0 & 0 & 0 & 0 & 0.7 & 0.25 & 0.05 & 0\\ 0 & 0 & 0 & 0 & 0 & 0.89 & 0.01 & 0.1\\ 0 & 0 & 0 & 0 & 0 & 0 & 0.98 & 0.02 \end{array}\right). \end{array}$$

Figure 3(b) shows a typical random network. The state transition probability matrix *P* is

(4) $$\begin{array}{c} \left(\begin{array}{cccccccc} 1 & 0 & 0 & 0 & 0 & 0 & 0 & 0\\ 0.1 & 0.02 & 0.88 & 0 & 0 & 0 & 0 & 0\\ 0 & 0.15 & 0.05 & 0.8 & 0 & 0 & 0 & 0\\ 0 & 0 & 0.15 & 0.25 & 0.6 & 0 & 0 & 0\\ 0 & 0 & 0 & 0.45 & 0.4 & 0.15 & 0 & 0\\ 0 & 0 & 0 & 0 & 0.75 & 0.24 & 0.01 & 0\\ 0 & 0 & 0 & 0 & 0 & 0.96 & 0.03 & 0.01\\ 0 & 0 & 0 & 0 & 0 & 0 & 0.99 & 0.01 \end{array}\right). \end{array}$$

Suppose that the same token's initial distribution is *u* = [0.25  0.25  0.15  0.15  0.1  0.05  0.04  0.01] and after a token random movement for 1000 steps, the state probability distribution for a scale-free network is [0.8287 0.0030 0.0198 0.0605 0.0675 0.0194 0.0011 0.0000], where, in 83% of cases, this token has reached the destination agent. On the other hand, the state probability distribution for a random network is [0.0171 0.0084 0.0575 0.3337 0.4904 0.0920 0.0009 0.0000], where, in only about 1.7% cases, this token has reached the destination agent. It proves what we observe in figures. The efficiency of information transmission in scale-free network is higher than in random network.

On the other hand, in teams, tokens do not simply move randomly from agent to agent when agents can build a better decision model [23]. Therefore, tokens are able to move faster to get accepted based on agents' local knowledge. To model that such movement is not completely random but is biased towards the target, we use a parameter *β* to make *P*(*s*_*i*_, *s*_*i*−1_) larger and *P*(*s*_*i*_, *s*_*i*+1_) smaller. However, this bias should be stronger as the token moves nearer to the target location because agents such as their neighbors are more likely to know the target. We model this by using *β*(*i*) = 1/*e* ^*α*_*i*_^. Informally, one can think of *β* as the total learning of the team about the team state and *α* as how much more agents “near” an agent know about it than agents “far” from it do. Using *α* and *β*, the Markov Chain state transitions can be rewritten as

(5) P˘(si,si−1)=P(si,si−1)+(1−β(i))P(si,si)+(1−2β(i))P(si,si+1),P˘(si,si)=P(si,si)−(1−β(i))P(si,si),P˘(si,si+1)=P(si,si+1)−(1−2β(i))P(si,si+1).

In addition, an *α* value of 0.97 is used to bias the links towards moving towards the target agent. Figures 3(c) and 3(d) show the effects on the scale-free and random distribution respectively. When tokens are especially close to the target, they are much more easier to get closer to the target and the agents will be the more efficient to deliver tokens.

Compared with the scale-free and random networks in random walk, if a token's initial distribution is the same as in the last section (i.e., *u* = [0.25 0.25 0.15 0.15 0.1 0.05 0.04 0.01], after 1000 movements), we will find that the state probability distribution is [1.0000 0.0000 0.0000 0.0000 0.0000 0.0000 0.0000 0.0000] and that the token will surely reach the destination. Moreover, after 100 moves, the state probability distribution is [0.9290 0.0029 0.0094 0.0242 0.0262 0.0078 0.0005 0.0000]. This means that, in 93% of cases, the token has reached the destination, which is better than token's random moves over 1000 steps in the same network topology. The theoretical analysis above verifies that the multiagent coordination will have a significant difference in different network topologies with both random and intelligent coordination strategies, so it will be an important factor we will consider in Simulator Design.

## 7. Simulator Design

**Figure 4:**
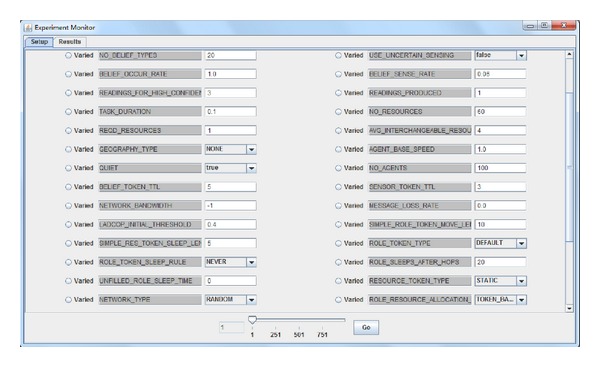
CoordSim allows a large number of parameters to be varied and also allows statistics to be recorded.

In this section, we describe the design of our simulator called CoordSim, shown as Figure 4. This simulator is capable of simulating the major aspects of coordination, including information sharing, task assignment, and resource allocation. CoordSim abstracts the environment by simulating only its effects on the system. Agents cannot receive any domain knowledge unless they sense it themselves or are “told” by a teammate over network. The physical resources required for tasks are simulated and allow only one agent to access them at any given time. There is no cost for transferring resources, and resources cannot be consumed or lost. The tasks are distributed randomly in an open environment. All agents are allowed to “think” and “act” at each time step, although the effects of their “actions” are abstractly simulated and they only take one time step.

Reward is simulated as being received by the team when a task is allocated to one agent. The most prominent advantage of CoordSim is that it allows a large number of parameters to be adjusted and also allows statistics to be recorded, such as the number of rewards and token movements. There are more than 20 parameters that can be varied, covering the major aspects of large heterogeneous coordination, and these configurations and records help to verify the validation of our approach.

### 7.1. System Architecture

**Figure 5:**
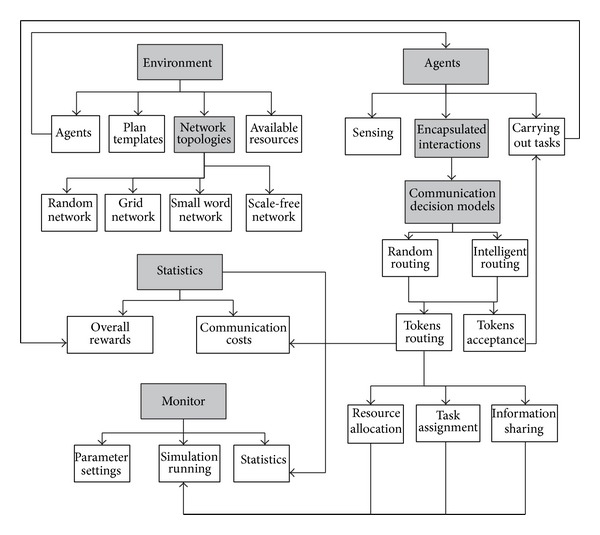
The architecture of CoordSim simulator.

The system architecture of CoordSim is illustrated as in Figure 5. It mainly consists of five modules: the environment, agents, communication decision models, as well as the monitor, and statistic model with user interface, which have been described in dark boxes. In environment module, it initiates the agents deployed in the environment, plan templates that could be activated to model agents' joint activities, the network that agents are originally connected, and the available resource for agents. According to the analysis of complex network effects in multiagent coordination, in our simulation, different topologies are taken into consideration, to allow users to simulate and analyze the influences of the network structures. The networked agents are organized as four typical network structures: random, grid, small world, and scale-free network. Please note that although we take the agents' mobility into consideration, the moving speed is far less than communication speed.

In the agent module, based on the knowledge of initial environment, the coordination processes include sensing environment, network interactions, and carrying out its own tasks. Especially, by encapsulating coordination into tokens, the network communication decision model is built with two algorithms: random routing and intelligent routing. Creating agents also includes the initialization of their heterogeneity. The key of the simulation is that agents initiate plans if they have gained enough information, either sensed or shared, and they may take different tasks and acquire required resources to achieve the system's common goal. Therefore, if the tokens encapsulated resources, tasks, or information are accepted by agents, they can perform the tasks with required resources and will credit the system with rewards. Otherwise, the token will not be stopped, and the agents will forward the tokens to the neighbor with communication costs.

### 7.2. Simulation of the Network

**Figure 6:**
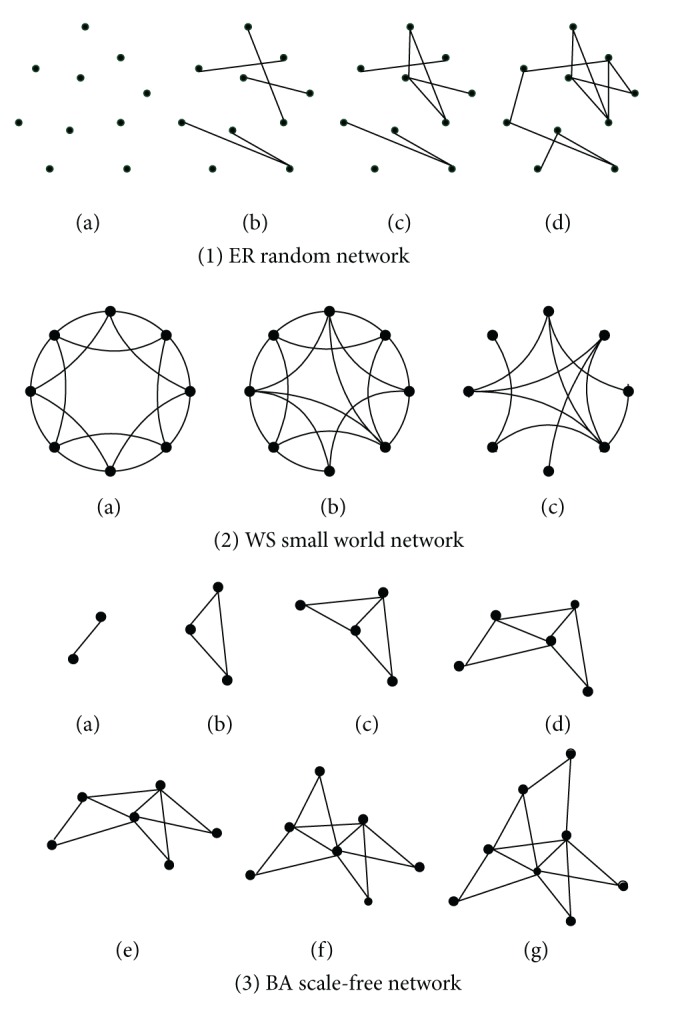
Process of building random network, small world network, and scale-free network.

In CoodSim, the network design is the most important part to network centric agents. Similar to human group where members typically maintain a small number of associates, when the system scales up, the network shows the attributes of the complex network. In this paper, we are mainly interested in the performances when networked agents are organized as four popular complex network structures: ER random network [25], grid network, WS small network [26], and BA scale-free network [21]. The network construction processes of setting up the organizations of *N* network agents with an average of *K* neighbors (for the algorithm design, *K* is always an even number) is shown in Figure 6. The total number of links in the network is *K* × *N*/2.

- ER random network: the network starts from nodes with empty links. For each time step, two nodes are picked up randomly and connected with a predefined probability *K*/(*N* − 1).
- Grid network: each node only connects with *K*/2 adjacent neighbors. The process of construction of grid network is straight forward as the one in [27].
- WS small world network: based on a regular grid network above, WS small world network is built with a relink process. In this process, every link in the network is selected with a probability *P*. If selected, one node of the link is kept but the other node will be replaced with a randomly picked node in the network.
- BA scale-free network: the network starts with a fully connected *K*/2 + 1 nodes. If the degree of each existing node *i* is *d*_*i*_, for each pending added node, the probability of linking it to the existing node *i* is *k*_*i*_/∑_*i*=1_^*m*^(*k*_*i*_).

In general, grid networks have the feature of clustering but do not present the small world effect [28]. The ER random network has small average path length, but it is not clustered relatively. The small world network is the transition from grid network to the ER random network, which has a small average distance and a high degree of clustering [29]. Because of the major hubs, the scale-free network exhibits strong robustness to failure, but sometimes it may weaken the performance of scale-free networks [30].

**Figure 7:**
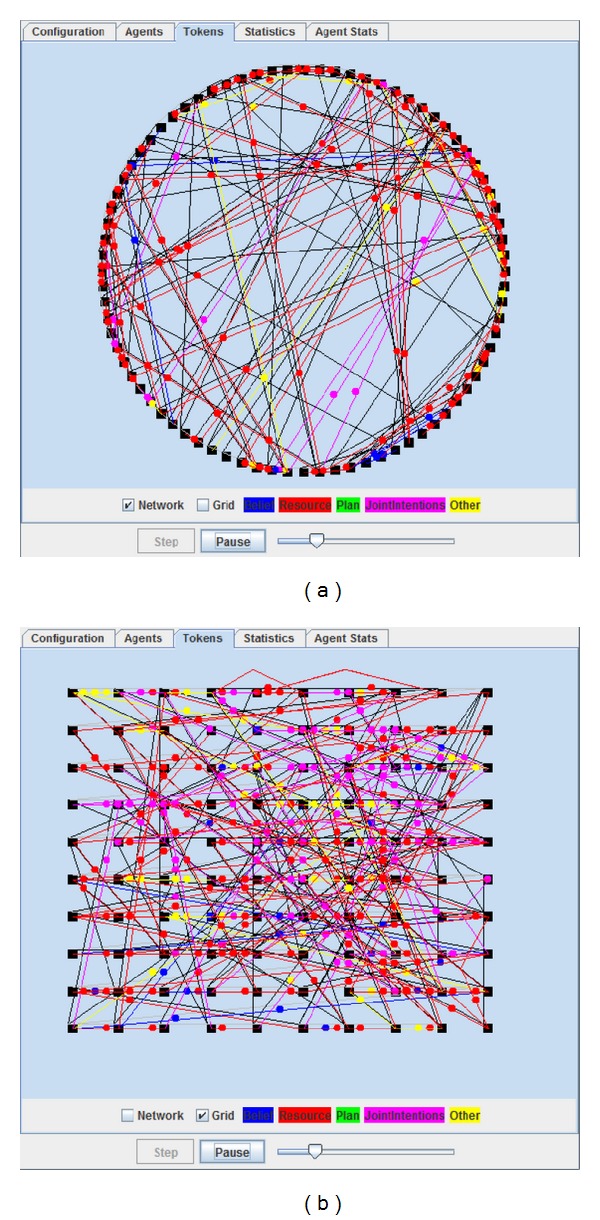
The interface for displaying interaction between networked agents.

In addition to the network design, we display the interaction between networked agents with a straightforward way which is shown in Figure 7.

### 7.3. Performance Evaluation and Statistics

Because of the complexity of the scalable-team coordination problem, there are many parameters that can be adjusted and tested. In our simulation, by varying more than 20 parameters and covering the major aspects of scalable coordination, we evaluate the system performance with both rewards and communication cost. Specifically, in this paper, we mainly interested in the parameters that contribute most to networked centric agents.

- If the communication cost is high, the team will receive a much lower reward when reach the same team goal.
- The communication processing rate is the number of tokens that an agent can pass per second. If there are more tokens, they have to be sent in the next second.
- Real-time control means that a task or a plan has to be carried out in a short period of time.
- Task importance describes how the reward is calculated. For example, the goal of a UAV team is to destroy as many enemy vehicles as possible, and the missing one is not a major concern. For USAR, however, missing one victim means that the team has failed, so every task and plan must be allocated.
- The team size is the number of agents in the team.
- A heterogeneous team describes the number of capability types among team members.
- The exclusive resource requirement describes the number of required resource types.
- Plan complexity defines the number of preconditions to activate each plan.

## 8. Simulation and Results

In this section, based on our simulator design, we briefly introduce the simulation of the networked multiagent system. The objective is to verify that our model is able to simulate the coordination of a few hundred networked agents.

### 8.1. Multiagent System Coordination

Because of the complexity of large-scale multiagent coordination, we typically choose four typical application domains: urban search and rescue (USAR), controlling wide area search munitions (WASMs) [31], RoboCup [32], and large agent team in strategy game and scheduling [33]. For example, in USAR, coordination is mainly focused on heterogeneous teams and there are only one or two tasks for each of plan templates. These domains are summarized according to the features listed in Table 1.

The experiment is to testify whether different complex network properties may influence the performance of multiagent systems with intelligent token routing algorithm in different application domains. Four complex network topologies have been tested and we have presented them in two groups of experiments. In the first group, we range the multiagent system size in each domain from 50 to 500 with on average 4 neighbors for each agent. In the second one, we fix the system size at 100 and the average number of associates for each agent varies from 2 to 10.

**Figure 8:**
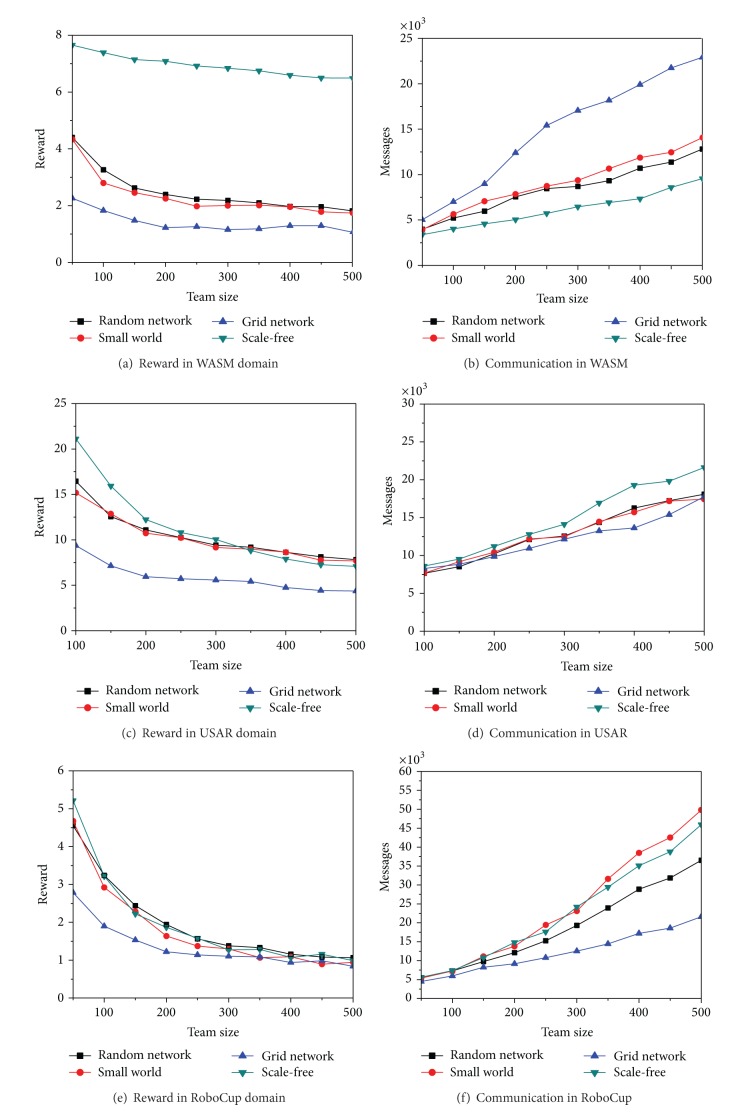

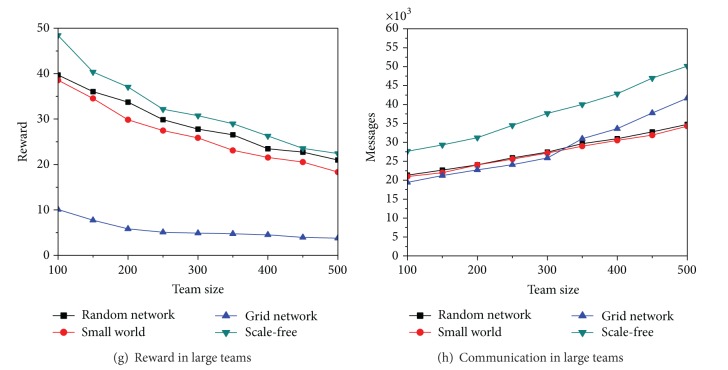
Effects of complex networks on different scale multiagent systems.

The first experiment's results are shown in Figure 8. In most domains, because of the property of the small world effect, the random network and the basic small world network outperform the grid-based network. In some domains, because of the property of the scale-free effect, the scale-free network may outperform the other three network topologies. The reason is that a few agents can act as hub nodes and obtain more knowledge of how to intelligently pass a given token in the right direction. On the other hand, in some domains such as RoboCup, the scale-free network produces more tokens and receives fewer rewards. The reason is that the hub nodes maintain many neighbors but the average tokens from each neighbor may be decreased. Therefore, the hub nodes cannot build a better model based on the previous tokens that they have received.

**Figure 9:**
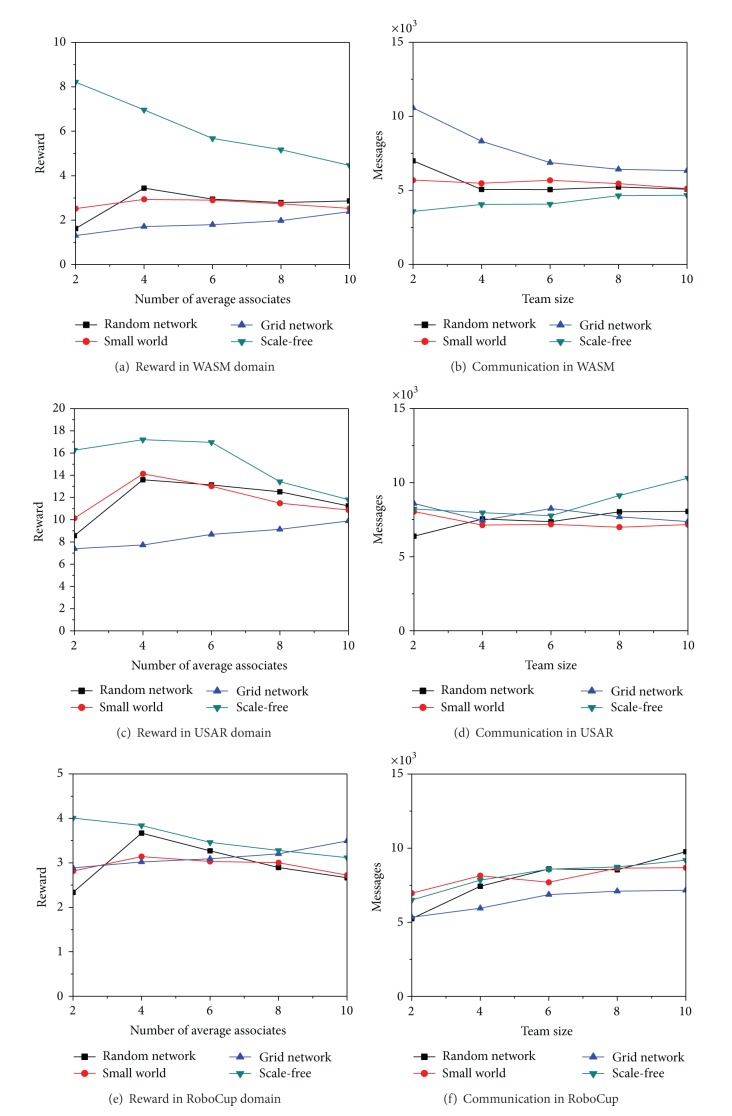

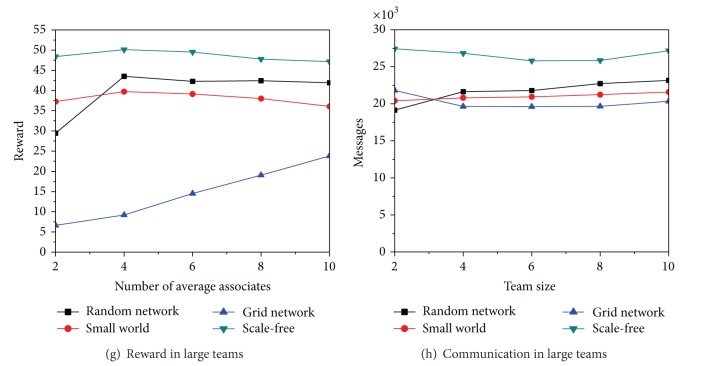
Effects of complex networks on different numbers of average associates.

The results of the second experiment are shown in Figure 9. On average, all of the network topologies increase their performance when agents keep an average of four neighbors instead of the average of two. But when the average number of neighbors increases except the grid network, the other network topologies' performances become worse. Therefore, when the number of neighbors per agent increases, the average number of tokens from each neighbor is decreased and the agents cannot maintain better decision models based on previously received tokens. This is most prominent in the hub nodes in a scale-free network that the scale-free network's performance decreased so quickly. Conversely, the grid-based network enhances its performance in two ways. First, the number of neighbors is a constant number for each agent. No agents will have a large number of neighbors. With the increasing number of neighbors, the average distance in the grid-based network is drastically decreased. In this way, it is much easier to intelligently pass tokens quickly to their destinations. Therefore, the small world effect is important on system efficiency.

### 8.2. Communication Transmission over Network

In the second simulation, we analyzed and simulated the communication transmission mechanism based on our designed probability decision model. In these experiments, we used a system with 400 agents and on average, each agent has four neighbors. One agent is randomly chosen as the source of a token *i* and another is randomly picked as the sink. The sink agent will firstly send out 20 related tokens *j* with *rel*⁡(*i*, *j*) and each will move MAX_STEPS = 50. Next, the source agent sends out token *i* and we measure how many tokens (communication costs) it takes to send *i* to the sink agent.

**Figure 10:**
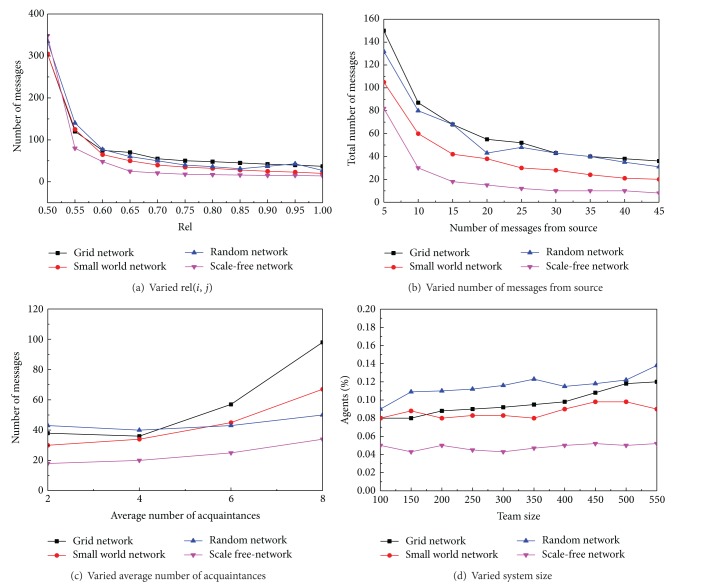
Communication transmission over different network topologies.

In Figure 10(a), we first varied the *rel*⁡(*i*, *j*) from 0.5 to 1. The efficiency of token transmission is improved in all networks with intelligent routing model rather than randomly passing (with *rel*⁡(*i*, *j*) = 0.5) with scale-free network having the best performance.

In Figure 10(b), we varied the number of related token *j* from the sink agent from 5 to 45. *rel*⁡(*i*, *j*) is set to 0.9. It shows that communications will become stable after the number of related token *j* is more than 15. This result also shows us that a few tokens are enough for agents to build local decision model. Scale-free network needs less communications as well.

In Figure 10(c), we run experiments with *rel*⁡(*i*, *j*) = 0.8 and each agent has on average 2 to 8 neighbors. It shows that more neighbors may not be helpful to improve network transportation with the same conclusion in Section 7.1.

In the last experiment shown in Figure 10(d), we ran experiments with different system sizes. The network transmission efficiency is measured as the percentage of agents involved for token passing:

(6) $$\begin{array}{c} \text{percentage}=\frac{\text{agents}\;\text{involved}\;\text{in}\;\text{token}\;\text{delivery}}{\text{Total}\;\#\;\text{of}\;\text{agent}\;\text{team}}. \end{array}$$

The experiment's results show that with different sizes of systems, the efficiency keeps steady. The percentage of random network changes slightly and its communication keeps high while scale-free network keeps least communication.

## 9. Summary and Future Work

In this paper, we presented a novel approach to model and simulate complex network attributes in a networked multiagent system with hundreds of agents. The key is that in typical large multiagent systems, sparsely distributed agents can only communicate directly with very few of the others and the network can be modeled as an adaptive complex network. By abstracting coordination and encapsulating it into tokens, we can model the interactions between networked agents and simulate them over the network. By building a simple local decision model on how to retransmit the coordination encapsulated in tokens, we can implement an integrated algorithm for decentralized agents to carry out the major aspects of coordination including sharing information, allocating tasks, and sharing resources; furthermore, we have taken a full account of the complex network effects and provide different topologies. All the designs have been encoded into our simulator CoordSim to test how the network structure affects the coordination performance. We discovered that in a typical networked system, small world effect uniformly helps the networked coordination, while scale-free effect by introducing high degree nodes may cut down the average distance of the network to help improve the performance but it may also result in the network congestion in the hub nodes to exacerbate the coordination efficiency.

While this work represents an important step towards simulating large decentralized networked multiagent system coordination, much work remains to be done. Critically in the simulation, we used the simplest instantiations of the algorithms, ignoring many performance enhancing techniques proposed in the literature which may be important to simulate real application domains. We intend to extend CoordSim to implement some of these extensions, for example, individual failures and agents' different mobility, which may impact performance in a large complex networked multiagent system.

## Figures and Tables

**Algorithm 1:**
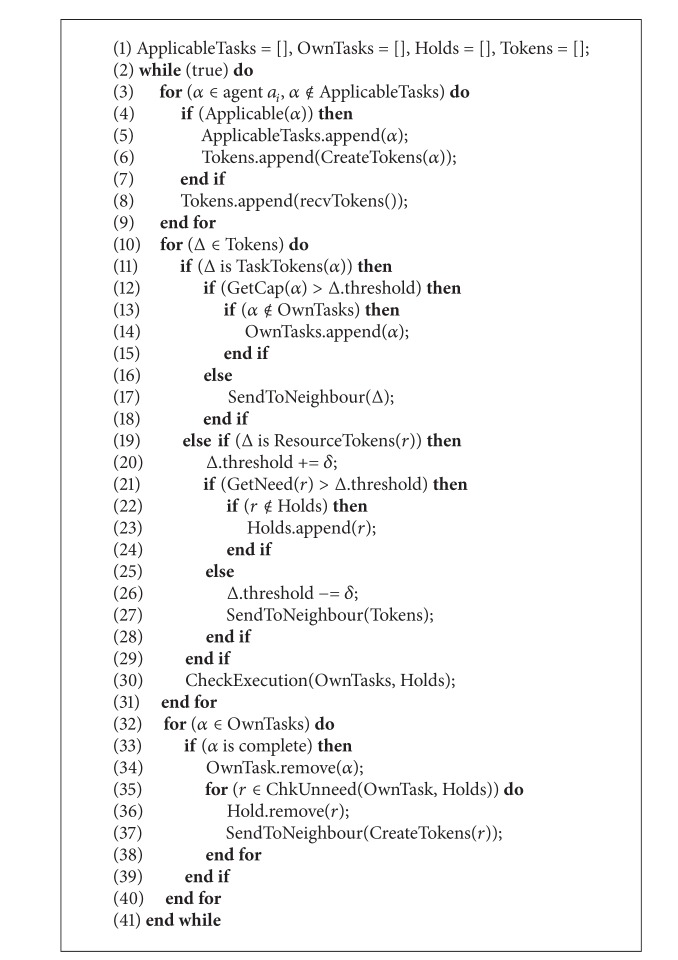
General process of agents' communication decision.

**Table 1:** Characters and settings of different application domains.

Scene	Team size	Plans	Task\plan	Res	Cap types	Info type
WASM	100	30	2	50	1	60
USAR	50	20	5	100	5	40
RoboCup	50	20	2	100	2	40
Large agent team	500	100	2	150	2	200
